# Nutritionally Driven Differential Gene Expression Leads to Heterochronic Brain Development in Honeybee Castes

**DOI:** 10.1371/journal.pone.0064815

**Published:** 2013-05-30

**Authors:** Lívia Maria Moda, Joseana Vieira, Anna Cláudia Guimarães Freire, Vanessa Bonatti, Ana Durvalina Bomtorin, Angel Roberto Barchuk, Zilá Luz Paulino Simões

**Affiliations:** 1 Departamento de Genética, Universidade de São Paulo-FMRP, Ribeirão Preto, São Paulo, Brazil; 2 Departamento de Biologia Celular, Tecidual e do Desenvolvimento, Instituto de Ciências Biomédicas, Universidade Federal de Alfenas, UNIFAL-MG, Alfenas, Minas Gerais, Brazil; 3 Departamento de Biologia, Universidade de São Paulo-FFCLRP, Ribeirão Preto, São Paulo, Brazil; University of Otago, New Zealand

## Abstract

The differential feeding regimes experienced by the queen and worker larvae of the honeybee *Apis mellifera* shape a complex endocrine response cascade that ultimately gives rise to differences in brain morphologies. Brain development analyzed at the morphological level from the third (L3) through fifth (L5) larval instars revealed an asynchrony between queens and workers. In the feeding phase of the last larval instar (L5F), two well-formed structures, pedunculi and calyces, are identifiable in the mushroom bodies of queens, both of which are not present in workers until a later phase (spinning phase, L5S). Genome-wide expression analyses and normalized transcript expression experiments monitoring specific genes revealed that this differential brain development starts earlier, during L3. Analyzing brains from L3 through L5S1 larvae, we identified 21 genes with caste-specific transcription patterns (e.g., *APC-4, GlcAT-P, fax, kr-h1* and *shot*), which encode proteins that are potentially involved in the development of brain tissues through controlling the cell proliferation rate (APC4, kr-h1) and fasciculation (GlcAT-P, fax, and shot). *Shot*, whose expression is known to be required for axon extension and cell proliferation, was found to be transcribed at significantly higher levels in L4 queens compared with worker larvae. Moreover, the protein encoded by this gene was immunolocalized to the cytoplasm of cells near the antennal lobe neuropiles and proximal to the Kenyon cells in the brains of L4 queens. In conclusion, during the larval period, the brains of queens are larger and develop more rapidly than workers’ brains, which represents a developmental heterochrony reflecting the effect of the differential feeding regime of the two castes on nervous system development. Furthermore, this differential development is characterized by caste-specific transcriptional profiles of a set of genes, thus pointing to a link between differential nutrition and differential neurogenesis via genes that control cell proliferation and fasciculation.

## Introduction

In the honeybee *Apis mellifera*, females that are fed on copious amounts of royal jelly throughout the larval development stages become queens, while those that receive smaller quantities of a mixture of glandular secretions, pollen and honey during the last two larval stages become workers. This initial feeding stimulus triggers a complex endogenous developmental program resulting in marked morphological differences between females (the queen and worker castes), representing one of the most famous examples of phenotypic plasticity in animals (see [1], [2]). Interestingly, contrary to what might be expected, the “underfed” workers exhibit a larger brain with more developed cognitive areas compared with the queens [3], which allows them to accomplish the variety of duties necessary to maintain a healthy colony, including foraging, nursing, nest cleaning and protection [3], [4]. In the initial stages of development, however, the brains of queen larvae appear to be larger than those of workers [5], implying that a high nutritional input may serve to drive nervous system development.

The adult insect brain exhibits a complex architecture composed of millions of glial cells, neurons and their respective tracts, forming structures known as neuropils that are organized to produce optic lobe, antennal lobe, central complex and mushroom bodies [6], [7]. As no neurogenesis occurs in adult honeybees [7], [8], all of the cells composing the bee brain must have originated by the end of post-embryonic development. Neuronal proliferation in the honeybee mushroom bodies (the integrative centers of the insect nervous system involved in learning and memory formation and responsible for processing olfactory information) begins at the first larval stage via symmetrical cell division in neuroblasts [9], [10]. Furthermore, Roat and Landim [5] showed that the queen brain presents a larger area corresponding to neuroblasts compared with workers during the last larval stage and the first half of pupal development. The enhanced differential neurogenesis favoring worker brain development is therefore expected to occur during the second half of the pupal period. Indeed, it has been observed that queen brain suffers far more cell death during the last part of pupal development than the worker brain [5], [9], [11], [12], which helps to explain the brain/body and mushroom body/brain size ratios characteristic of adult workers [3].

The study of the impact of different diets on nervous system development represents a broad area of biological research; most of the published results on this topic come from experiments conducted with rodents. For example, Matos et al. [13] showed that maternal malnutrition results in learning deficits and a predisposition to anxiety and depression in offspring, due to a decreased number of hippocampal neurons, which are known to proliferate during early life. Thus, the differential feeding regime experienced by presumptive queen and worker larvae represents a natural experiment and provides good model for the identification of genes linking differential nutrition to differential nervous system development. This model can therefore help to improve our understanding of the molecular and cellular processes involved in caste development in social insects as well as those related to the outcomes of early-life malnutrition in other animal models.

Herein, we describe the morphological characteristics of the honeybee brain as it develops during larval stages in both castes, including the higher rate of cell proliferation in the brains of queens. Additionally, we present a list of genes obtained via oligonucleotide microarray hybridization that are differentially expressed between queen and worker brains during the fourth larval stage as well as the transcriptional profiles of ten genes associated with neurogenesis: *anaphase promoting complex 4* (*APC-4*), *galactosylgalactosylxylosylprotein 3-beta-glucuronosyltransferase P* (*GlcAT-P*), *failed axon connection* (*fax*), *krüppel homolog –1* (*kr-h1), short stop* (*shot*), *ataxin*-*2* (*atx-2*), *cryptocephal* (*crc*), *Ephrin Receptor* (*ephR*), *dachshund* (*dac*), and *tetraspanin 5D* (*tsp5D*). All but the first two genes were previously identified through cDNA hybridization experiments as being differentially expressed between castes in whole-body preparations of fourth instar honeybee larvae (L4, [1]) or were found to be expressed only in neurons during *Drosophila* development (*kr-h1,* [14], [15]), indicating that they might participate in the differential brain morphogenesis between *A. mellifera* castes. Using anti-shot *in situ* hybridization combined with immunostaining, we demonstrate up-regulation of *shot* gene expression in queen brains compared with those of workers. Our results point to *shot* as a key player in the differential brain morphogenesis induced by differential feeding in honeybee larvae.

## Materials and Methods

### Bees and Brains

*A. mellifera* larvae were collected from colonies (Africanized hybrids) at the Experimental Apiary of the University of São Paulo at Ribeirão Preto, Brazil. Larvae of the same age were obtained as described in Barchuk et al. [16]. The developmental stages were classified according to the criteria proposed by Michelette and Soares [17] and Rembold et al. [18] (see Table 1).

**Table 1:** Developmental stages and characteristics of the *A. mellifera* larvae used in this work.

Dev stage	Characteristics	Hours after hatching
**L3**	0.0015–0.004 g (W)/0.0013–0.007(Q)	35–55
**L4**	0.004–0.0248 g (W)/0.004–0.044 g (Q)	56–80(W)
**L5F2**	0.06–0.11 g (W)/0.09–0.18 g (Q)	95–105(W)
**L5S1**	Larval period after sealing (spinning) (W/Q)	115–130(W)

Modified from Michelette and Soares [17] and Rembold et al. [18]. L3 =  third larval instar; L4 = fourth larval instar; L5F2 = second feeding phase of the fifth larval instar; L5S1** = **first spinning stage of the fifth larval instar. Q = queens. W = workers.

Samples were obtained from third instar larvae (L3), fourth instar larvae (L4), fifth instar, second feeding phase larvae (L5F2), and first spinning phase larvae (L5S1) to be used for gene transcription quantification. These samples consisted of 3 pools of 10 brains each, which were dissected under sterile 0.9% NaCl solution. The brains were then immediately transferred to TRIzol® reagent (Invitrogen) and frozen at −80°C until total RNA isolation was performed. For whole-mount preparations, 10–15 brains were dissected in ice cold sterile NaCl solution (0.9%) and fixed.

### Phalloidin/DAPI Staining

Brain whole-mounts were prepared for phalloidin and DAPI staining according to Ashburner [19], with some modifications. Briefly, the brains were dissected in ice cold sterile NaCl solution (0.9%), fixed in 1∶1 formaldehyde (16%) and N-heptane (Merck) for 1 day at 4°C and permeabilized in 0.5% Triton X-100 in PBS (PBT). Subsequently, a 1∶500 dilution of phalloidin (Rhodamine Phalloidin Conjugate – Molecular Probes) was added to the solution containing the brains, followed by incubation for 20 minutes and then several washes with phosphate buffered saline with 0.1% Triton-X (PBT). This preparation allowed us to visualize the track of axons. In the last wash, the brains were incubated with DAPI (4′,6-diamidino-2-phenylindole, dihydrochloride, 1∶2,000; Sigma) for 4 minutes, washed with PBT, mounted in 80% glycerol and analyzed using a Leica TCS-SP5 Scanning Confocal Microscope (Leica Microsystems).

### Cell Proliferation Assay using EdU

The brains of L5 workers and of queens were dissected in sterile 0.9% NaCl, then incubated for 3 hours in a solution containing culture medium (larval honeybee medium, LHB [20]) and 40 mM 5-ethynyl-29-deoxyuridine (EdU, Click-iT TM EdU Imaging Kits – Invitrogen) at room temperature under constant gentle shaking. The brains were then fixed in 1∶1 formaldehyde (16%) and N-heptane (Merck) for 30 min and subsequently transferred to the Click-iT TM EdU Imaging Kit reaction mixture (43 mL 10X reaction buffer; 38 mL distilled water; 20 mL copper sulfate; 1.2 mL Alexa Fluor 594; 50 mL reaction buffer additive), followed by incubation for 30 min. The brains were washed 5 times for 5 minutes each with PBT 0.5%, and nuclei were then stained with 1∶1,000 Hoechst for 30 minutes under shaking. Another 5 washes were performed, and the brains were mounted in 80% glycerol (Merck). The obtained images were analyzed using a Leica TCS-SP5 confocal microscope (Leica Microsystems).

### *FISH* - Fluorescent *in situ* Hybridization

#### Generation of FISH probes

Oligonucleotide probes were generated from PCR amplicons that contained a T7 RNA polymerase promoter at the 5′ end. Transcription reactions were performed using the FISH Tag RNA Kit (Invitrogen; [21]), following the manufacturer’s suggested protocol. The amplified fragment of the *shot* gene was 396 bp, and the following primer sequences were employed (5′ to 3′):

*shot* (FWD) GGA GGA GTT GTT GTC GTG GT.

*shot* (REV) CGC CAA TCT TCC CAA CTA AA.

*shot* (T7+FWD) **TAA TAC GAC TCA CTA TAG GGC GA** G GAG GAG TTG TTG TCG TGG T.

*shot* (T7+REV) **TAA TAC GAC TCA CTA TAG GGC GA** C GCC AAT CTT CCC AAC TAA A.

#### Whole-mount FISH protocol

Our whole-mount FISH protocol followed the method of Saunders & Cohen [22] for *Drosophila* ovaries. Brains were dissected in Ringer saline (NaCl 0.17 M, KCl 0.01 M, CaCl_2_ 0.003 M) and fixed for 20 minutes using 1 mL N-Heptane, 160 µL HEPES (0.1 M HEPES – pH 6.9; 2 mM MgSO_4_; 1 mM EGTA), 40 µL of 20% paraformaldehyde (PFA) and 20 µL DMSO. Then, the brains were washed rapidly two times with 100% methanol and one time with 100% ethanol, then stored. The brains were rehydrated through a series of washes using methanol/PBS+Tween-20, 0.1%, and fixed again for 20 minutes using 80 µL of PBS+0.1% Tween-20, 100 µL of 4% PFA, and 0.2 µL of 0.1% Triton X-100. After the post-fixation step, 20 µg/mL proteinase-K was added to the solution for 1 minute, and the samples were then pre-incubated in hybridization solution at 45°C for 1 hour, followed by incubation for 16 hours at 45°C with a fluorescent RNA probe under shaking (hybridization solution: 25 mL formamide 50%; 10 mL 4x SSC; 500 µL 1x Denhardt’s solution; 2.5 mg heparin 50 µg/mL; 500 µL Yeast tRNA (Invitrogen); 500 µL salmon testes DNA (Sigma).

### Immunocytochemistry

For anti-shot immunostaining, fixed brain whole-mounts were washed 4 times in 0.5% PBT and incubated in a blocking solution (5% goat serum and 0.1% BSA in PBS plus 0.5% Triton X-100) for 1 h and for 16 h in a solution with 1∶200 anti-shot mAbRod1 (Developmental Studies of Hybridoma Bank). An Alexa Fluor 488 goat anti-mouse antibody (Molecular probes) at a 1∶200 dilution was used as the secondary antibody. The negative control was incubated without the primary antibody (see Figure S1). Washing steps were performed using 0.5% Triton X-100 in PBS, and DAPI (4′,6-diamidino-2-phenylindole, dihydrochloride, Sigma) staining was conducted at room temperature for 4 min, followed by another washing series in PBS with 0.5% Triton X-100. The brains were then mounted in 80% glycerol and analyzed using a Leica TCS-SP5 scanning confocal microscope (Leica Microsystems). Quantification of nuclei in developing brains and nuclei of proliferating cells was done using the plugin Particle Analysis - Nucleus counter at ImageJ (W. S. Rasband, National Institutes of Health, Bethesda, MD; http://rsbweb.nih.gov/ij/plugins/itcn.html), with default parameters.

### Quantification of Gene Transcription

#### Oligonucleotide microarray hybridization

Microarray experiments were performed and are described according to the Minimum Information About a Microarray Experiment (MIAME) specifications [23], and the obtained data have been deposited in the Gene Expression Omnibus database (GEO, at the NCBI database) under accession number GSE39239.

Total RNA isolated from the brains of fourth larval stage queens and workers was purified using the RNA Cleanup kit (RNeasy Mini Kit, QIAGEN), and 1 µg of total RNA was then used for the amplification procedure with the Amino Allyl MessageAmp™ II aRNA Amplification Kit (Ambion). A 6 µg sample of amplified RNA was subsequently labeled with Cy3 or Cy5 dye (RPN5661, GE Healthcare). Two sets of labeled probes were then hybridized to whole-genome oligonucleotide microarrays acquired from the Functional Genomics Unit of the W.M. Keck Center at the University of Illinois at Urbana-Champaign through Dr. Gene Robinson. The slide design was based on the design of the INDAC long oligo set for *Drosophila melanogaster*, including 12,915 unique oligos (http://www.biotech.uiuc.edu/functionalgenomics). Prior to pre-hybridization, each slide was UV cross-linked and plunged into 0.2% SDS, washed with water, plunged into ethanol and centrifuged at 2000 *g* for 3 min. Pre-hybridizations were carried out for at least 45 min in a warm solution (42°C) containing 20% Formamide, 10% Denhardt’s solution (50x), 33.2% SSC 20x, 0.1% SDS and 0.5% tRNA (10 mg/mL) and then rinsed in Milli-Q water, plunged into isopropyl alcohol and dried via centrifugation at 2000 *g* for 3 min. Hybridizations were carried out with dye-swaps. The probes (in 60 µL of 49% formamide, 49% SSC 20x and 0.2% SDS) were preheated at 50°C for 3 min and then transferred to slides and covered with lifter-slip cover glasses (22×60, 31.25 µL). The slides were subsequently placed in single-slide hybridization chambers (CLS2551, Corning) and incubated in a water bath to allow hybridization to occur for 17 h at 42°C. The washing procedure included the following steps: 2×SSC and 0.1% SDS; 2×SSC; 0.1×SSC; and Milli-Q water. Each washing step was conducted for 3 min at room temperature, except for the first step, which consisted of 10 sec at 42°C. The slides were dried via centrifugation at 2000 *g* for 2 min and scanned using an Axon Genepix 4000B scanner (Molecular Devices) with GenePix® Pro 6.0 software (Agilent Technologies, Santa Clara, CA) at a 10 micron resolution, during which Cy3 was excited with a green laser (532 nm) and Cy5 with a red laser (635 nm).

#### Bioinformatics analysis

Images from hybridized slides were processed using GenePix® Pro 6.0 software with the default parameters. All normalizations and fold change calculations were performed using functions of the *Limma* library of the R/Bioconductor package (R Development Core Team, 2012, [http://www.R-project.org]), as described in Barchuk et al. [1]. Briefly, the background was corrected by adding to the background intensities a positive constant (offset correction = 50), which restrains spurious variation in log-ratios, particularly at low intensity spots. The “print-tip loess” normalization was used to correct for within-array dye and spatial effects and single channel normalization was used to facilitate comparison between arrays. Subsequently, the log_2_ ratio (Queen’s sample intensity/Worker’s sample intensity) was determined for each probe in each array. After applying Bayesian statistics to shrink estimated standard errors, we used the moderated *t*-statistic test for each gene. Following statistical analyses (adjusted *p*<0.05; *B*>0) and removal of values from spots with low intensity signals (compared to empty spots), a list of 16 differentially represented transcript sequences (DRTs) was obtained. All DRTs with *Drosophila* orthologs were identified using the protein domains database Pfam (http://pfam.sanger.ac.uk/search) and Gene Ontology analysis (http://www.geneontology.org/).

All ten genes selected for RT-qPCR analysis were identified in the honeybee genome (assembly version 4.0) available at the Baylor College of Medicine website (http://www.hgsc.bcm.tmc.edu/content/honey-bee-genome-project) and manually annotated using the Artemis platform [24]. The characteristics of the deduced amino acid sequences were assessed using tools available at www.justbio.com, www.cbs.dtu.dk, www.expasy.ch and www.ncbi.nlm.nih.gov/sites/gquery?itool=toolbar. Domains of predicted proteins were also identified using Pfam Search. The primers for qPCR were designed using Primer 3 (Rozen, 2000, Primer3 on the WWW for general users and for biologist programmers; see Table 2) and Primer Express v2.0 (Applied Biosystems).

**Table 2:** Characteristics of the primers used in the RT-qPCR assays.

Primer pairs	Predicted gene	Sense primer	Antisense primer	Annealing temp (°C)	Fragment length (bp)
***atx-2***	GB18802	ACAACATCCCAACAGTCAC	TGTAGGTCGCAAAGGTAATGG	60	162
***APC-4***	GB18982	TGGAGGAAGAAAGGGAGTGA	TTGTTTCACGATAAGCGGATG	60	151
***crc***	GB19338	GGAGATGTGGAAGCTTGTCA	ATGGTTGTACTGGTTGTAAAGT	60	133
***dac***	GB17219	GCACCTCAGTCACATGCAAT	GACATGTTCGGGTTCACCTT	62	150
***EphR***	GB12585	ACCAACGCAACCGTGATCC	ATAGCGTGAGGCGTCTTCTT	60	152
***Fax***	GB17380	TCAGCACGTGCTGGGTACT	AACCGAATTGGGACACCTCT	60	140
***Kr-h1***	GB14867	GCACTGGCAGTGACAAGGAA	GTGGAGTGTTATCGTAAGTAGCAA	60	76
***shot***	GB17469	TTCAGGGAAACGGCTTGGAA	GTGCCGACACCAGTCAGG	60	160
***Tsp5D***	GB14751	CTGCGGCGTGCGGGATTA	TTGTTACGCCCCTCGGAGT	60	129
***GlcAT-P***	GB12549	GGATTGAAGTTCGAGCATCTG	CTCTGATCCATTGTAAGCCAC	60	111
***rp-49***	AF441189	CGTCATATGTTGCCAACTGGT	TTGAGCACGTTCAACAATGG	60	150
***β-actin***	AB023025	TGCCAACACTGTCCTTTCTG	AGAATTGACCCACCAATCCA	60	156

#### Quantitative assays via Real-Time PCR (RT-qPCR)

Total RNA was isolated from each pooled sample (10 brains) using 500 µL of TRIzol® following the manufacturer’s protocol (Invitrogen) and incubated in the presence of RNase-free DNase (Promega) to eliminate contaminating DNA. First-strand cDNA was synthesized via reverse transcription using 1 µg of total RNA, SuperScript II reverse transcriptase and an oligo (dT 12–18) primer (Invitrogen), as described by Nascimento et al. [25].

Comparisons of the gene transcription profiles between queens and workers were conducted via real-time PCR using SYBR® Green mix and the 7500 Real Time PCR System (Applied Biosystems). The reactions were carried out in a 20 µL mixture containing 10 µL of 2x SYBR® Green Master Mix (Applied Biosystems), 0.8 µL of each 10 mM gene-specific forward and reverse primer (Table 2) and 1 µL of the first-strand cDNA sample diluted 1∶5 in water. The PCR conditions were as follows: 50°C for 2 min and 95°C for 10 min, followed by 40 cycles of 95°C for 15 s and 60°C or 62°C (*dac*) for 1 min. Each of the three pooled samples was analyzed in triplicate, and the transcripts of the *rp-49* (*ribosomal protein 49*) and *β-actin* genes were used as a reference (geometrical means; [2], [26], [27]). PCR efficiency values (E) were calculated from the slope obtained after running standard curves using the formula E = 10^(−1/slope)^−1 (Table S1). The relative level of each gene transcript was calculated using the 2^−ΔΔCT^ method (Applied Biosystems User Bulletin #2; [28]). Statistical analyses were carried out with SigmaStat 3.1 software (Jandel Corporation, San Rafael, CA, USA) using two-way ANOVA with two-tailed probabilities, followed by the Holm-Sidak *post hoc* test. RT-qPCR experiments were performed according to the Minimum Information for Publication of Quantitative Real-Time PCR Experiments (MIQE) guidelines [29].

## Results

### Differential Brain Morphogenesis During Larval Caste Development in *A. mellifera*

Despite our understanding of the organization of the adult honeybee brain, little is known about its development during larval instars. To investigate morphological differences in *A. mellifera* brains in the context of caste differentiation, we performed high-resolution neuroanatomical studies of whole-mount preparations of larval brains from the third to the middle of the fifth instar (L5S1). This is a critical period in which larvae experience a caste-specific hormonal environment as a result of the switch in the feeding program [1], [30].

**Figure 1:**
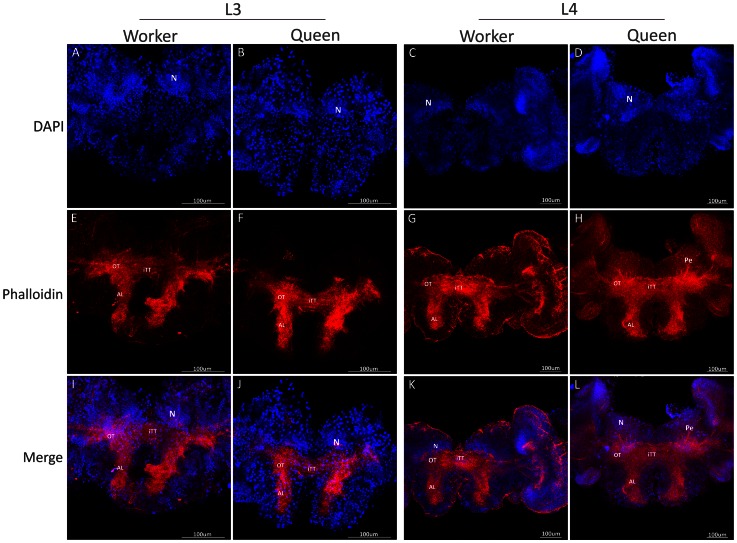
Frontal view of the central neuropils in the 3^rd^ and 4^th^ larval instars of *A. mellifera* castes. Queen and worker brains are highlighted with phalloidin (red) and DAPI (blue). In these stages, there are clusters of neuroblasts (N) on each side of the brain (**A–D**). Phalloidin stains axons from optic tubercles (OT), inter-tubercle tracts (iTT) and the antennal lobe (AL) (**E–L**). No morphological differences can be observed between the castes at the 3^rd^ stage, whereas in the 4^th^ stage, the peduncles (Pe) of mushroom bodies start to develop in queen brains (**H, L**).

**Figure 2:**
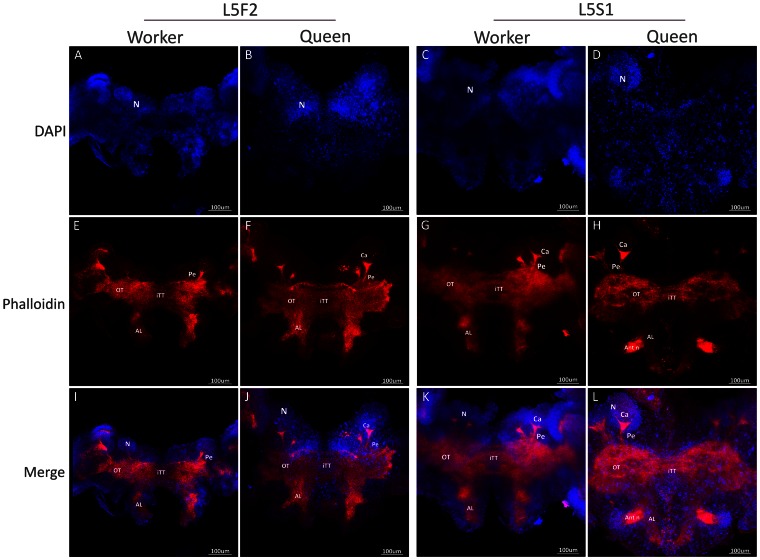
Frontal view of the central neuropils in the 5^th^ larval instar of *A. mellifera* castes. Queen and worker brains during the L5F2 and L5F1 larval instars are highlighted with phalloidin (red) and DAPI (blue). In L5F2, there are calyces (Ca) of mushroom bodies visible in queen brains (**E, I**), which are not seen in workers until the S1 stage (**H, L**), as well as nerves forming at the antennal lobe in queen brains (**G, K**). The same structures are less well developed in workers. N: neuroblasts, OT: optic tubercles, AL: antennal lobe, Ant n: antennal nerve, iTT: inter tubercle tracts.

**Figure 3:**
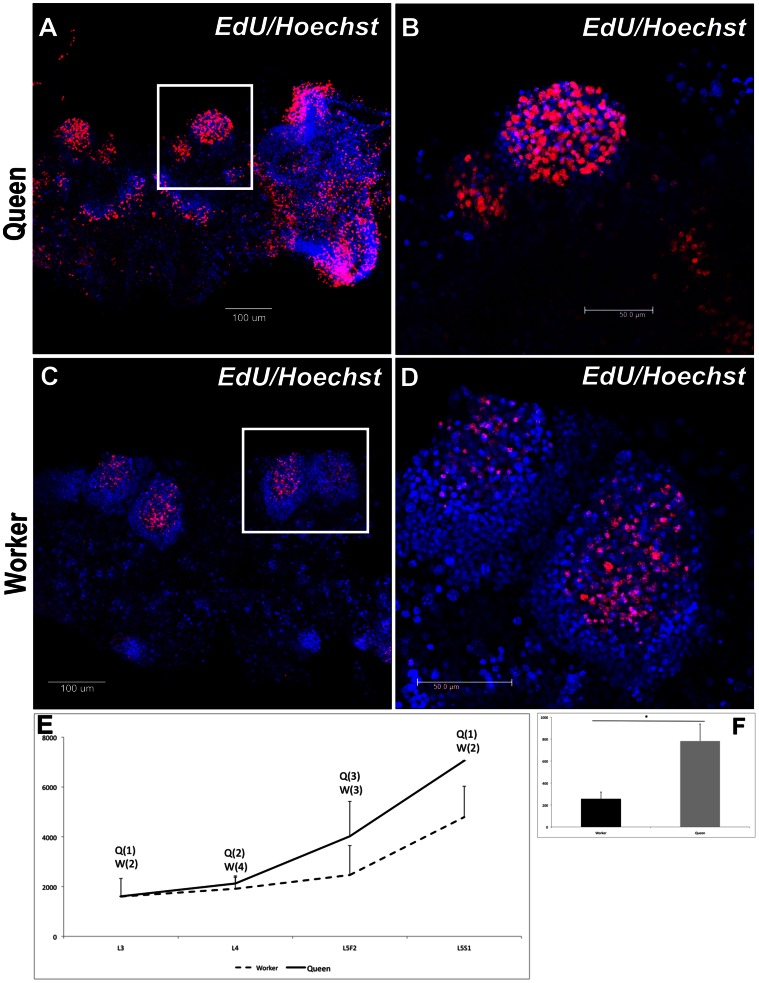
Cell proliferation in brains of queen and worker honeybees. The queen brain shows a larger area of cell proliferation compared with the worker brain during the 5^th^ instar. (**A**) A queen brain stained with EdU-Alexa fluor 594, showing increased cell proliferation in mushroom bodies. (**B**) Details of cell proliferation in the mushroom bodies of queens. (**C**) A worker brain stained with EdU-Alexa fluor 594 (red) shows that it is mainly the cells in the mushroom bodies that are proliferating. (**D**) Details of the cell proliferation in the mushroom bodies of workers. Blue: Hoechst staining (to visualize nuclei); red: EdU-Alexa fluor 594 staining (EdU binds to DNA during cell division). (**E, F**) Quantification of nuclei in developing brains (E) and nuclei of proliferating cells (F; *p*<0.05). L3, L4∶3^rd^ and 4^th^ larval instars, respectively; L5F: fifth larval instar (feeding stage); L5S: fifth larval instar (spinning stage).

**Figure 4:**
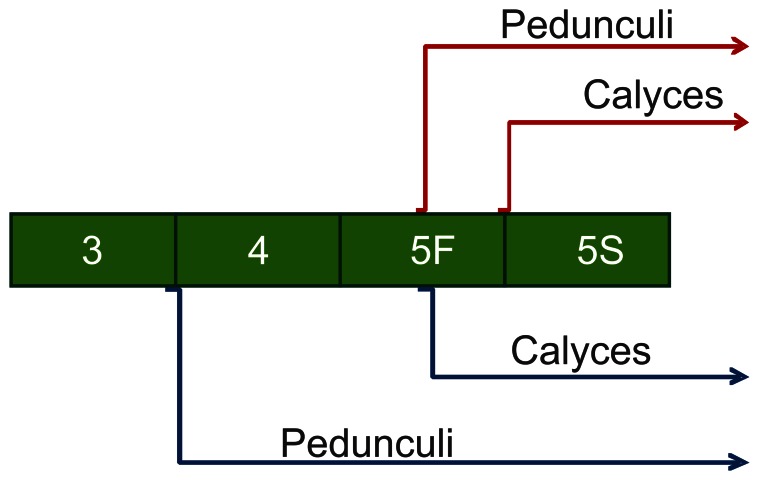
Scheme of the developmental timing of the mushroom body neuropil regions in *A.* *mellifera* workers and queens. During brain development, calyces and pedunculi appear earlier in queens, indicating that the queen brain develops more rapidly than that of workers, affecting the synchronization of brain development between castes. L3, L4∶3^rd^ and 4^th^ larval instars, respectively; L5F: fifth larval instar (feeding stage); L5S: fifth larval instar (spinning stage); blue arrows: queens; red arrows: workers.

In third instar larvae (L3), DAPI staining showed early clusters of neuroblast nuclei on each side of the brain (Figure 1 A, B), and phalloidin staining revealed axons projecting from the optic tubercles, antennal lobes and inter tubercle tracts (Figure 1 E, F, I, J). In this stage, no morphological differences were detected between queens and workers. In fourth instar larvae (L4), phalloidin staining showed the first sign of the formation of mushroom body structures, i.e., the pedunculus, in the brain of queen larvae (Figure 1 G, K), whereas this structure was still absent in workers at this stage (Figure 1 H, L). As the mushroom bodies continued to develop, the shape of the cluster of DAPI-stained nuclei changed, becoming more organized in the apical region of the brain (Figure 1 C, D). These clusters will subsequently form the distinctive Kenyon cell population [10]. In addition, the neuropiles that form calyces became more organized, first appearing during L5F2 in queens (Figures 2 E, I; 4). In workers, the development of these structures was delayed. The formation of peduncles was detectable only in developmental stage L5F2 (Figures 2 F, J; 4), and calyces appeared later, during the early spinning stage, L5S1 (Figures 2 H, L; 4). Interestingly, during this period of development, there were axon bundles belonging to the antennal nerve observed in the antennal lobes of queens (Figures 2 G, K; 4). We also showed that the queen brain presented a larger area of cell proliferation compared with the worker brain during the L5F2 developmental stage, as visualized following EdU staining (Figure 3). In this stage, positive EdU staining was observed in most of the mushroom bodies (and in the whole brain) in the brains of queens (Figure 3 A, B), but only in the center region of the mushroom bodies in workers’ brains (Figure 3 C, D). Moreover, the mushroom bodies in the queen brains showed a higher proportion of cell proliferation, as indicated by the density of pink dots (Figure 3 B, D, F). Nuclei quantification of whole brains showed increasing number of cells from L3 to L5S1, and higher numbers in queens’ brains from L4 (Figure 3 E; see also Figure 4).

### Differentially Expressed Transcripts in the Brains of L4 Queens and Workers Revealed by Oligonucleotide Hybridization

Because the morphological differences between queen and worker brains begin at L4 (see Table 1 and the previous section), which is the developmental stage characterized by the greatest differences in JH titers between castes [30], likely in a response to the differential food intake [31] that occurs during larval development, we performed oligonucleotide microarray hybridization analyses comparing RNA samples from the brains of queens and workers during this post-embryonic stage. This approach allowed us to identify genes associated with the differential brain development detected between castes. Following stringent statistical criteria (adjusted *p*<0.05; *B*>0; and removal of values from spots with low intensity signals), we obtained a list of 16 differentially represented transcript sequences (DRTs) in honeybee fourth instar larval brains, 12 of which were up-regulated in queens and 4 in workers. Of these genes, 5 and 2, respectively, have *Drosophila melanogaster* orthologs and represent coding sequences (Table 3). Three DRTs in queens (BB170027A10E09.5, BB170032B20H12.5, BB160007B10F02.5) and one in workers (DB746346) did not match any known protein coding gene in *A. mellifera*. Based on predicted functions and functions demonstrated in other biological systems, the products of the up-regulated transcripts found in queens are associated with cell proliferation, fasciculation or interneuronal connections [e.g., *GlcAT-P* (GB12549), *APC-4* (GB18982), *dp* (GB11706), α-*Cat* (GB12545) and *gmd* (GB19686)]. The DRTs that were over-represented in the worker brain samples are genes whose products have been demonstrated to function during the development of eye cells and GABAergic neurons [*Aristaless Related Homeobox* (GB16661)] and in brain cells in response to stressors [*PP2C* (GB10931)].

**Table 3:** Differentially represented sequences obtained via microarray hybridization of samples from the brains of L4 workers and queens of *A. mellifera*, clustered by Pfam database.

Official_set_ID	Name	Flybase_ID	ORF (pb)	Product (aa)	Scaffold_ID(Group)	Accession number (XP_)	Pfam	Fold change
Q: GB11706		CG33196	8196	2731	6.26	623961.2	PWWP superfamily	3.79
Q: GB12021	*PR Domain*		2961	986	4.27	396029.4	SET superfamily	4.64
Q: GB12545	*α-catenin*	CG17947	2730	909	15.29	625229.1	Vinculin family	5.28
Q: GB12549	*GlcAT-P*	CG6207	1140	379	11.34	394932.3	Glycosyltransferase family A	5.55
Q: GB15759	*Sodium-coupled monocarboxylate transporter 2-like*		1788	595	1.64	394160.2	SSF superfamily	5.57
Q: GB18914			600	199	9.21	1121927.2	*zinc ribbon* family	5.85
Q: GB18982	*APC4*	CG32707	2175	724	Un.537	393301.3	Apc4 superfamily	3.36
Q: GB19686	*gmd*	CG8890	1077	358	7.26	395164.3	NAD dependent epimerase/dehydratase family	3.34
Q: GB19966	*Y+L amino acid transporter 2-like*		1467	488	15.21	395239.4	Amino acid permease	3.75
W: GB10931	*PP2C*	CG17746	990	329	Un.537	623418.2	PP2C	3.80
W: GB11875			558	185	Un.696	3251929.1	–	4.90
W: GB16661	*Aristaless Related Homeobox*	CG2819	753	250	14.23	1121339.1	Homeodomain superfamily	5.39

W = Worker. Q = Queen. Name = Gene name.

### Transcriptional Profiling of Genes Associated with Neurogenesis During the Larval Development of Honeybee Queens and Workers

Due to our interest in identifying genes underlying differential brain development in response to differential feeding in honeybees, using RT-qPCR, we assessed the expression profiles of ten genes linked to neurogenesis (Table 4) during larval development (L3-L5S1, see Table 1 and “Bees and brains” from the M&M Section). Some of these genes were identified in the oligonucleotide microarray hybridization analyses described above (*GlcAT-P* and *APC-4*), while others were identified as differentially expressed between castes via cDNA microarray hybridization analyses of the whole bodies of fourth instar honeybee larvae, and are associated with neurogenic processes (*tsp5D*, *dac*, *fax, crc*, *atx-2, EphR* and *shot*; [1]) or are known to be linked to neurogenesis in different animal models (*kr-h1;* [32], [33]).

**Table 4:** Characteristics of genes whose expression was analyzed by RT-qPCR.

Gene	ORF (bp)	Protein (aa)	Mol Weight (kD)	Pfam	Accession number
***GlcAT-P***	1140	379	43.35	Glycosyltransferase family A	XP_394932.3
***APC4***	2175	724	81.97	Apc4 superfamily	XP_393301.3
***tsp5D***	918	306	29	Tetraspanin family	XP_394297
***dac***	2346	781	83	SKI/SNO/DAC family	XP_394482
***fax***	1176	391	44.7	Outer mitochondrial membrane transport complex protein	XP_393141
				Glutathione S-transferase, C-terminal domain	
***crc***	1074	357	41.3	bZIP transcription factor	XP_393709
***atx-2***	2733	911	100.4	LsmAD domain	XP_392675
***EphR***	2991	996	110	Ephrin receptor ligand binding domain	XP_392034
				GCC2 and GCC3	
				Fibronectin type III domain	
				Protein tyrosine kinase	
				SAM domain (Sterile alpha motif)	
***shot***	15480	5159	581.3	Plectin repeat	GB17469
				Spectrin repeat	
				EF hand	
				Growth-Arrest-Specific Protein 2 Domain	
***Kr-h1***	1500	500	56.8	C2H2 zinc finger	NM_001242470 (Gene ID 100576395)

**Figure 5:**
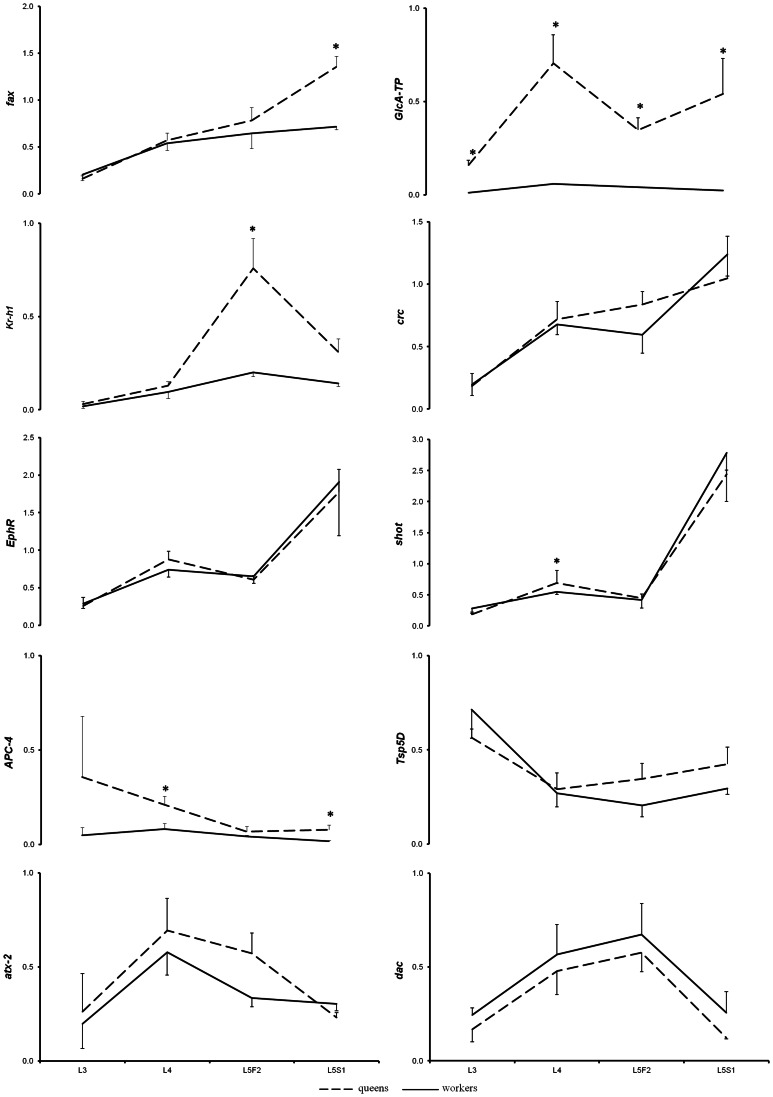
Transcription profile of genes associated with neurogenesis in the brains of *A.* *mellifera* females of different castes. The ordinates represent the relative transcript levels assessed by RT-qPCR. The data were normalized to *ribosomal protein-49*. Three biological samples were analyzed in technical triplicates. L3, L4, L5F and L5S: larval stages; *: statistically significant differences between castes (two-way ANOVA, *p*<0.05); *fax*: *failed axon connection; GlcAT-P*: *galactosylgalactosylxylosylprotein 3-beta-glucuronosyltransferase P*; *kr-h1*: *krüppel homolog –1*; *crc: cryptocephal; EphR: Ephrin Receptor*; *shot: short stop*; *APC-4*: *anaphase promoting complex 4*; *tsp5D*: *tetraspanin 5D*; *atx-2*: *ataxin*-*2*; *dac*: *dachshund*.

As shown in Figure 5, all ten genes were found to be transcribed in the brains of queens and workers throughout the entire period analyzed. Our results revealed three groups of expression patterns. One was represented by the majority of the genes, which showed a general increasing trend from the 3^rd^ to the spinning stage of the 5^th^ larval instar in both castes (*fax*, *GlcAT-P, kr-h1, crc*, *EphR* and *shot*). Interestingly, *crc* was found to be expressed at an 8-fold higher level in the heads than in the brains in both castes (data not shown), supporting the idea that it plays specific roles in larval head development [34]. The second group of expression profiles corresponds to the *APC-4* and *tsp5D* genes, which presented the opposite trend, exhibiting decreasing transcription levels. The third group included *dac* and *atx-2*, which showed the highest levels in L4-L5F2. Five of the genes mentioned above (*APC-4, fax, GlcAT-P, kr-h1* and *shot*) were differentially transcribed between castes (*p*<0.05) during at least one of the studied developmental stages, all of which showed higher expression in queens.

### The Shot Gene is Expressed Earlier and at Higher Levels in the Brains of Queens

**Figure 6:**
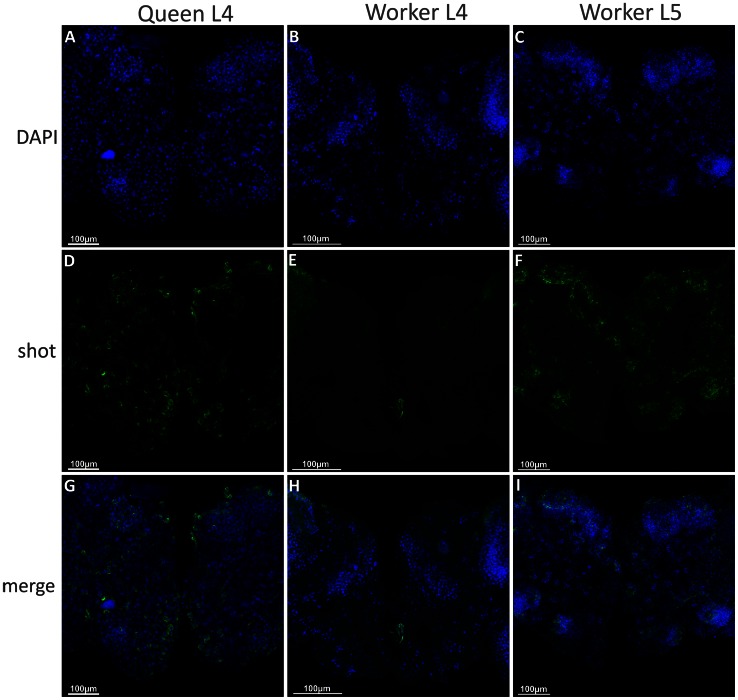
*In situ* hybridization to detect *shot* expression in the brains of *A.* *mellifera* workers and queens. Confocal images of brain of 4^th^ instar honeybee queens show *shot* mRNA throughout the entire organ (**A, D, G**). No mRNA was detected in worker brains in the 4^th^ instar (**B, E, H**), and expression only appeared in the 5^th^ stage (**C, F, I**). Green: shot mRNA; blue: DAPI. Note the *shot* mRNA surrounding nuclei.

**Figure 7:**
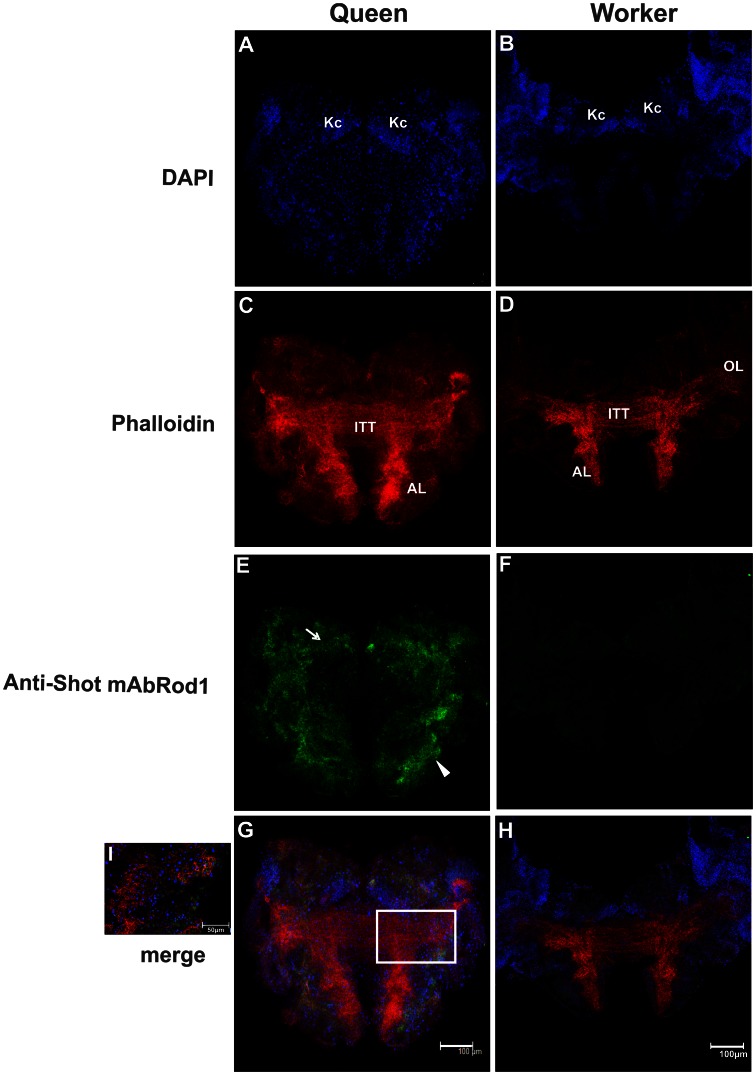
Shot immunostaining during the 4^th^ larval instar of *A.* *mellifera* castes. Triple labeling with DAPI (A, B), phalloidin staining (**C, D**) and anti-shot (mAbRod1; **E, F**). (**G**) Shot protein was immunolocalized to the cytoplasm of cells near the antennal lobe neuropiles (arrowhead) and proximal to the Kenyon cells in the brains of L4 queens (arrow). (**H**) As observed for mRNA, Shot protein was absent in worker brains in the 4^th^ larval instar. Confocal plane of a queen brain (**I**) showing Shot staining in the cytoplasm of cells (arrows and arrowheads).

In addition to its higher transcription rate demonstrated via RT-qPCR, using *in situ* hybridization, we were able to detect *shot* gene transcripts in L4 brains of queens, but not in workers (Figure 6 D, G, E, H), in which *shot* transcripts could only be detected during L5F2 (Figure 6 F, I). Moreover, *shot* mRNA was shown to be translated at a higher level in the brains of queens than in workers (Figure 7). Anti-shot immunostaining (using the mAbRod1 antibody) detected Shot protein in the cytoplasm of cells near the antennal lobe neuropiles and proximal to the Kenyon cells in the brains of L4 queens (co-localized with actin filament bundles in some regions) (Figure 7 G, I), whereas no positive images were obtained when workers’ brains were processed (Figure 7 F, H).

## Discussion

### Heterochronic Larval Brain Development in Honeybee Castes

Our results show that pedunculi, calyces, and antennal lobes are more developed in queen than in worker larvae. Thus, because we also demonstrated the occurrence of higher rates of cell proliferation in queens, both processes, proliferation and fasciculation, appear to be responsible for the observed differential morphogenesis of female brains between honeybee castes. A larger area corresponding to neuroblasts in the brains of queens than in workers’ was also reported by Roat and Landim [5] based on the examination of histological sections from the last stage of larval development. Similarly, Groh and Roessler [35], reported heterochronic shifts in the development of the olfactory centers during pupal development for queens and workers. However, our data not only show that the brains of queen larvae are more developed, but also that brains of queens develop faster than those of workers, thus representing a heterochronic reflex associated with differential feeding leading to differential nervous system development in female honeybees (Figure 4).

### Patterns of Gene Transcription and their Putative Involvement in Differential Brain Development in Honeybee Castes

With the aim of identifying the molecular mediators of the differential feeding regime related to the differential brain morphogenesis observed between castes, we employed the oligonucleotide microarray hybridization approach to compare RNA samples from the brains of queens and workers at L4 and RT-qPCR to determine the transcription profiles of selected genes during the critical period of caste differentiation, from L3-L5, which represents the majority of the larval phase [17], [18]. A total of 24 genes (16 detected via microarray hybridization and 8 via qPCR) showed significant levels of transcription during the larval development of queens and workers, which suggests that their respective protein products participate in brain development in honeybees.

*PP2C*, which was one of the genes up-regulated in the worker brains (detected via microarray hybridization), belongs to a family of phosphatase genes that are involved in the regulation of stress-activated protein kinase cascades, which relay signals in response to external stimuli [36]. In honeybees, the protein encoded by this gene might intracellularly transduce the shift in the feeding regime observed in worker larvae during the L3 stage (see [1]), thus controlling the expression of downstream genes, resulting in a restriction of worker brain development during the larval phase. The other gene with a *Drosophila* ortholog that was up-regulated in the worker brain is the predicted *Aristaless-Related Homeobox* (*Al-related*) gene. In vertebrates, some members of the Aristaless family (group II) are involved in optic system development [37] and in the proliferation and differentiation of GABAergic neurons. Because *A. mellifera* workers exhibit more facets in their compound eyes and GABAergic neurons play essential roles in olfactory memory and sensory integration, an up-regulation of Al-related proteins would allow the development of nervous structures that are fundamental for key skills in the adult bee, such as navigation and communication.

Among the differentially expressed genes that were up-regulated in the brains of queens, *GlcAT-P* (homolog of the mammalian glucuronyltransferase *b3gat1*) and *APC-4* deserve special attention. GlcAT-P glucuronyltransferase activity is required for proteoglycan and glycoprotein biosynthesis, which is important for the development and function of the central and peripheral nervous system [38]. In *Drosophila*, GlcAT-P is responsible for the growth of peripheral nerves during larval development [39]. Our microarray data showed that *GlcAT-P* was transcribed at a 5.55-fold higher level in the brains of queens than in workers during the L4 stage (Table 3). This result was confirmed by RT-qPCR, which also showed higher levels of gene transcription in the brains of queens throughout the larval period (*p*<0.05; Figure 5). The increased GlcAT-P activity observed throughout larval development could explain the greater and more rapid fasciculation observed in the brains of queens. The other gene, *APC4*, which encodes a member of the protein complex that regulates cell cycling and dendrite-axon morphogenesis [40], is expressed at a 3.36–higher level in the brains of queens. Interestingly, RT-qPCR confirmed this finding and showed that this gene is transcribed at a higher level in the brains of queens during the last larval instars (*p*<0.05 for L4 and L5S1; Figure 5), when we could also detect more cell proliferation in queen brains. The protein products of these two genes might be involved in the mechanisms leading to the differential brain development observed between honeybee castes through controlling the cell proliferation rate (APC4) and fasciculation (GlcAT-P).

*Fax*, which was one of the genes that showed an increasing transcription profile and was differentially expressed between castes, was initially characterized based on mutations that enhanced *Abelson tyrosine kinase* (*abl*) mutant phenotypes [41]. Subsequent studies showed that Fax interacts with different proteins involved in axon pathfinding [42]. The genomic sequence of *Amfax* encodes a putative protein of ∼45 kD, similar to two of the largest and one of the first Fax protein variants found in *D. melanogaster* [41]. However, the deduced amino acid sequence of *AmFax* does not show stretches of hydrophobic amino acids, thus indicating that it is unlikely to play a role in cell-cell interactions, which are processes in which the *D. melanogaster* ortholog was suggested to participate [41]. Like *kr-h1*, *fax* is up-regulated in the brains of queens during the 5^th^ larval instar, suggesting that it participates in the differential brain morphogenesis observed between castes, likely enhancing fasciculation and interneuronal connections.

The *Kr-h1* gene encodes a member of the zinc finger transcription factor family implicated in neural morphogenesis and the regulation of gene expression in response to ecdysteroids [43], [44]. The transcription profile of this gene in developing brains shows low levels in both castes during L3-L4 and high levels only in queens during the 5^th^ larval instar, particularly in L5F2. In spite of showing a negative correlation with neuronal morphogenesis in developing *D. melanogaster* mushroom bodies [43], the observed differential transcription of this gene suggests that Kr-h1 plays a caste-specific role in brain morphogenesis during most of larval development in honeybees. Because this developmental phase is characterized by premetamorphic ecdysteroids peaks and *kr-h1* expression has been suggested to be regulated by the ecdysone signaling, the up-regulation of *kr-h1* in the brains of queens may be a response to the higher titers of ecdysteroids in this caste as a consequence of differential feeding [1], [30], thus contributing to shaping the development of a larger brain queen larvae.

### The Role of Shot Expression in the Differential Larval Brain Development between Castes

The other gene that was differentially expressed between castes, *Shot,* encodes cytoskeleton-associated proteins with binding sites for F-actin and microtubules that is required for sensory and motor axon extension in *D. melanogaster* [45], [46]. The *shot* gene was initially identified based on a mutation in which embryonic motoneurons fail to reach their targets [47] and the correct axonal pathfinding of mushroom body neurons does not occur [48], but it has also been suggested to be involved in cell proliferation [49]. These studies showed that clusters of neuroblasts homozygous for a mutant form of *shot* exhibit significantly reduced cell numbers. Because we observed higher rates of cell proliferation and fasciculation in the brains of queens, the higher expression of *Amshot* in fourth instar queen larvae, as indicated by RT-qPCR, in *situ hybridization* and immunostaining, suggests that this gene is a pivotal player in the gene expression cascade induced by differential feeding in honeybees, which may underlie the differential brain morphogenesis that occurs in castes of *A. mellifera*.

In conclusion, we showed that queen larvae develop larger brains than worker larvae and that the queen brain develops more rapidly than that of workers, thus representing a form of developmental heterochrony, reflecting the effect of the differential feeding regime of the two castes on nervous system development. We also showed that this differential brain development is characterized by caste-specific transcriptional profiles for a set of genes (*APC-4, GlcAT-P, fax, kr-h1* and *shot*), similar to the classically proposed explanation for the brain/body weight ratio favoring humans compared to chimpanzees [50], [51]. In particular, based on diverse experimental approaches, we showed that the *shot* gene is more highly expressed in the brains of queens. These results point to a link between differential nutrition and differential neurogenesis, via genes that control cell proliferation and fasciculation, thus highlighting a biological question with more general, biomedical implications. Since it has been previously demonstrated that epigenetic events mediate variations in nutritional inputs leading to bifurcating developmental trajectories in *A. mellifera* [52], we will next address the methylation pattern of the found differentially expressed genes, and conduct functional assays, especially to shed light on the extent of the role of these genes in differential neurogenesis.

## Supporting Information

Figure S1 Negative control for the immunolocalization of Shot (mAbRod1) in brains of honeybee queens at developmental stage L4. (**A**) DAPI; (**B**): phalloidin/rhodamine; (**C**) incubated only with the secondary antibody (Alexa-fluor 488); (**D**) merge.
(TIF)

Table S1 Slope, R^2^ and efficiencies values for each pair of primers used.
(DOC)
