# Supplementary figures and images for: Nutritionally Driven Differential Gene Expression Leads to Heterochronic Brain Development in Honeybee Castes
Source: PLoS One. 2013 May 30;8(5):e64815. doi: 10.1371/journal.pone.0064815 (PMC3667793; doi:10.1371/journal.pone.0064815)

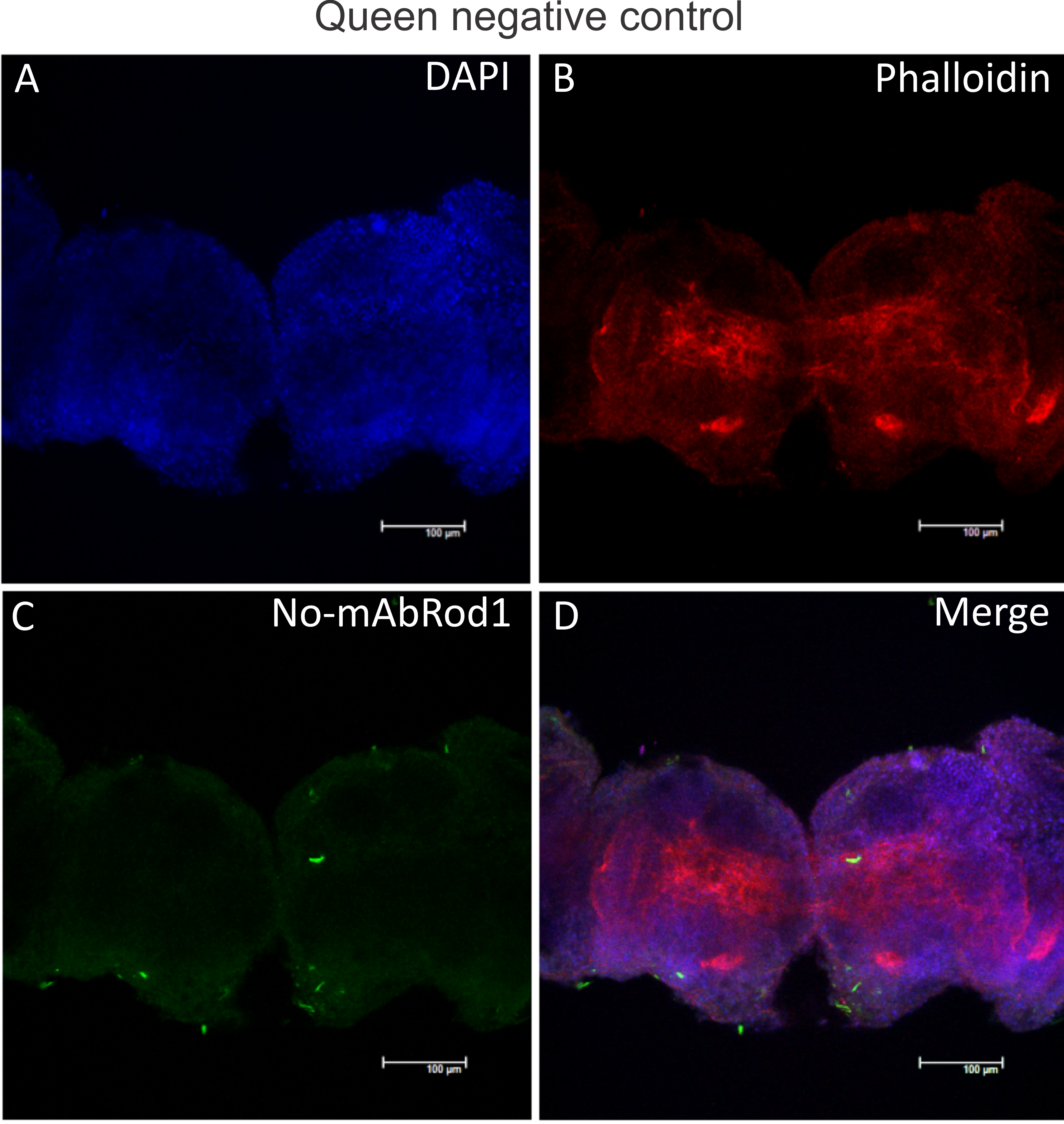

Supplement: Figure S1 — Negative control for the immunolocalization of Shot (mAbRod1) in brains of honeybee queens at developmental stage L4. (A) DAPI; (B): phalloidin/rhodamine; (C) incubated only with the secondary antibody (Alexa-fluor 488); (D) merge. (TIF) [file pone.0064815.s001.tif]
