# Supplementary material for: Nutritionally Driven Differential Gene Expression Leads to Heterochronic Brain Development in Honeybee Castes
Source: PLoS One. 2013 May 30;8(5):e64815. doi: 10.1371/journal.pone.0064815 (PMC3667793; doi:10.1371/journal.pone.0064815)
Supplement: Table S1 — Slope, R2 and efficiencies values for each pair of primers used. (DOC) [file pone.0064815.s002.doc]

| *Primers* | Slope | R^2^ | Efficiency (%) |
| --- | --- | --- | --- |
| *APC-4* | -2.1889 | 0.9934 | 186.3192 |
| *atx‐2* | ‐3.306692 | 0.996432 | 100.6398 |
| *crc* | ‐3.452379 | 0.999311 | 94.8298 |
| *dac* | ‐3.266464 | 0.992632 | 102.3678 |
| *Dom* | -2.3938 | 0.9952 | 161.6651 |
| *EphR* | ‐ 3.277474 | 0.988389 | 101.8892 |
| *fax* | ‐ 3.754766 | 0.988559 | 84.6410 |
| *GDP* | -2.2012 | 0.9975 | 184.6411 |
| *Glcat* | -2.1462 | 0.9992 | 192.3747 |
| *kr-h1* | -3.3292 | 0.9988 | 99.6974 |
| *PP2C* | -3.1945 | 0.9921 | 105.6071 |
| *shot* | ‐ 3.953762 | 0.993551 | 79.0291 |
| *Tsp5D* | ‐ 3. 691185 | 0.995442 | 86.6017 |
